# Youth Aware of Mental Health for Danish School Adolescents: Study Protocol for a Cluster‐Randomized Feasibility Pilot Trial

**DOI:** 10.1002/mpr.70064

**Published:** 2026-03-09

**Authors:** Lasse K. Harris, Michella Heinrichsen, Marie Petri, Hanne Elberling, Julie L. Forman, Merete Nordentoft, Annette Erlangsen, Britt R. Morthorst

**Affiliations:** ^1^ Child and Adolescent Mental Health Center Copenhagen University Hospital ‐ Mental Health Services CPH Copenhagen Denmark; ^2^ Danish Research Institute for Suicide Prevention ‐ DRISP Mental Health Center Copenhagen Copenhagen Denmark; ^3^ Department of Clinical Medicine Faculty of Health and Medical Sciences University of Copenhagen Copenhagen Denmark; ^4^ Section of Biostatistics Department of Public Health Faculty of Health and Medical Sciences University of Copenhagen Copenhagen Denmark; ^5^ Copenhagen Research Center for Mental Health Mental Health Centre Copenhagen Copenhagen Denmark; ^6^ Centre for Mental Health Research National Centre for Epidemiology and Population Health The Australian National University Canberra Australia; ^7^ Department of Mental Health Johns Hopkins Bloomberg School of Public Health Baltimore Maryland USA

**Keywords:** adolescent, feasibility trial, suicide prevention, youth aware of mental health

## Abstract

**Objectives:**

(1) To investigate the feasibility, acceptability, and fidelity of delivering the Youth Aware of Mental Health (YAM) program in Danish public schools to 8‐9th‐grade students, and (2) to explore the preliminary effects of the program on self‐reported outcomes at 3‐ and 6‐month follow‐up.

**Methods:**

This two‐armed, cluster‐randomized feasibility pilot trial involves 8–10 public schools across Denmark. School 8‐9th‐grade students are allocated to receive either the YAM program in addition to curriculum as usual or curriculum as usual plus YAM posters. Primary outcomes are participation rates, program adherence, and questionnaire completion. Secondary outcomes are assessed through questionnaires measuring suicidal behavior, psychological distress, well‐being, help‐seeking intentions, and suicide‐related stigma. Data are collected at baseline, 3‐month, and 6‐month follow‐up and analyzed using descriptive statistics and multi‐level models to estimate preliminary effects.

**Results:**

Recruitment began in August 2024, and the 6‐month follow‐up assessment will conclude in July 2025.

**Conclusion:**

This trial provides insight into the practical delivery of YAM in Danish public schools. Findings will inform potential adaptations and guide future implementation designs for school‐based mental health strategies.

## 1 Introduction

Adolescence represents a crucial period for mental health development. Mental distress, including symptoms of depression and anxiety, is highly prevalent among youth worldwide (Polanczyk et al. 2015; Patel et al. 2007). These concerns are frequently reported during early adolescence and tend to peak in late adolescence and early adulthood, with individuals aged 16–24 consistently showing higher rates of poor mental well‐being than any other age group (World Health Organization 2021; Kessler et al. 2005; Gore et al. 2011).

Among the most troubling signs of adolescent mental distress is deliberate self‐harm (DSH), which may or may not involve suicidal intent (O’Connor and Nock 2014). A common precursor to DSH is suicidal ideation. Approximately 4% of community sample adolescents aged 12–15 years report experiences of suicidal ideation, with prevalence estimates rising to over 15% in mid‐adolescence (Madge et al. 2008; Larsson and Sund 2008). In Norway, around 2% of adolescents reported suicidal ideation within the last 2 weeks (Larsson and Sund 2008). First episodes of DSH are often recorded between the ages of 14 and 16 (Nock 2010; Gillies et al. 2018). Overall, rates and severity of DSH tend to increase throughout this developmental period (Gillies et al. 2018). In Denmark, the highest rates of hospital‐recorded DSH were found among young adults aged 15–24 (Morthorst et al. 2024, 2016). However, the true figures are likely to be higher; for every hospital‐recorded suicide attempt, 2‐ and 6‐fold as many female and male adolescents, respectively, reported suicide attempts without seeking medical attention (Danielsen et al. 2024). Together, these trends underscore the need to intervene.

Media and digital platforms are increasingly recognized as influential factors in shaping adolescent attitudes, including toward DSH and suicide. Systematic reviews revealed that depictions of suicidal behavior in news reports, entertainment media, and online forums are associated with increased suicide‐related outcomes, particularly among young people (Niederkrotenthaler et al. 2020, 2019; Haw et al. 2013). High‐profile cases, such as the Netflix release of *13 Reasons Why* and peer discussions on unregulated social media platforms (e.g., Reddit, Discord, Snapchat, Instagram, Bluesky), were linked to imitation behavior and increased psychological distress among vulnerable adolescents (Haw et al. 2013; Ayers et al. 2017; Ruder et al. 2011). Furthermore, these platforms often enable continuous exposure to cyberbullying, which can intensify feelings of isolation and distress (Hinduja and Patchin 2010). Given adolescents' developmental susceptibility and the largely unmoderated nature of online content, it is critical that interventions help foster media literacy, promote healthy coping strategies and help‐seeking behavior, and strengthen resilience against harmful digital influences.

Given nearly 50% of youth who self‐harm form the decision within an hour, that is from ideation to the act itself (Madge et al. 2008; Deisenhammer et al. 2009), it is important that preventive efforts reach adolescents before risk escalates. Systematic reviews have identified several effective strategies within school settings, particularly psychoeducational programs, which improve emotional health literacy, raise awareness of mental health issues, including suicidal behavior, and foster peer support networks (Calear et al. 2016; Robinson et al. 2013; Zalsman et al. 2016; Pistone et al. 2019). Interventions, such as the Good Behavior Game (Wilcox et al. 2008), Signs of Suicide (Aseltine et al. 2007), and Youth Aware of Mental Health (YAM) (D. Wasserman et al. 2015), have demonstrated significant reductions in suicidal ideation and DSH in randomized controlled trials.

The YAM program was evaluated as part of the SEYLE trial, including 11,110 adolescents across 10 European Union countries. In that study, 2721 adolescents received the YAM intervention, which demonstrated significant reductions in suicidal ideation and DSH at 12‐month follow‐up compared to a control group receiving curriculum as usual and YAM educational posters (D. Wasserman et al. 2015). However, subsequent trials in the U.S. and Australia reported mixed findings (Lindow et al. 2020b; McGillivray et al. 2021), highlighting the need for context‐specific evaluations. YAM targets all adolescents, regardless of individual risk level, and is well‐suited for public school settings where entire age cohorts can be reached. To our knowledge, YAM has not yet been scientifically evaluated in a Nordic school context. This trial therefore aims to address this gap by examining the program's delivery and short‐term effects on 8‐9th‐grade students.

## 2 Objectives

This trial has two aims:

- *Aim I:* To investigate the feasibility, acceptability, and fidelity of delivering the YAM program in Danish public schools to 8‐9th‐grade students, assessed by (1) program participation and response rates, (2) adolescent endorsement, and (3) adherence to the intervention manual.
- *Aim II:* To explore the preliminary effects of the YAM program on self‐reported mental health outcomes at 3‐ and 6‐month follow‐up provided to 8‐9th‐grade students in Danish public schools.

## 3 Methods

### 3.1 Design and Setting

**FIGURE 1:**
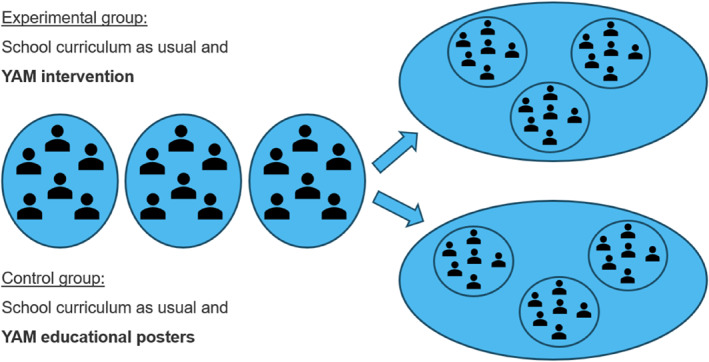
YAM RCT flowchart.

This study is conducted as a two‐armed, cluster‐randomized feasibility pilot trial evaluating the delivery of the YAM program in 8‐9th‐grade students in Danish public schools. Cluster randomization is performed at the school level. Participating schools are randomly assigned to either receive (a) the YAM program, in addition to their standard curriculum, or (b) the standard curriculum plus YAM educational posters displayed in the classrooms (Figure 1). The trial is conducted among students aged 14–16 years in 8–10 public schools across Denmark to ensure geographical and demographic diversity. Data are collected at baseline and at 3‐ and 6‐month of follow‐up. The protocol was preregistered on ClinicalTrials.gov (ID: NCT06549764).

### 3.2 School Recruitment and Eligibility

Schools are recruited through targeted outreach via municipal Child and Youth Departments and school administration offices, as well as through collaboration with the World Health Organization (WHO)‐led Healthy Cities Network via Local Government Denmark and national networks within education and health. The recruitment strategy is designed to ensure a geographically diverse representation and to maximize participation by public schools whose students are likely to represent general adolescent populations and, thus, improve the external validity (C. Wasserman et al. 2012). Participating schools are asked to designate a contact person to assist with administrative tasks if possible.

### 3.3 Enrollment and Consent Procedures

**FIGURE 2:**
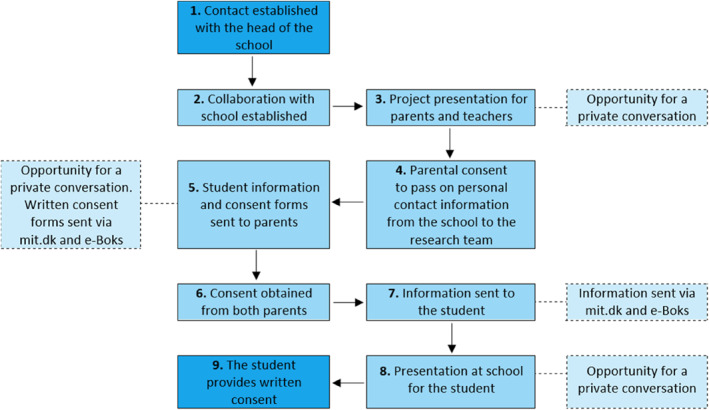
YAM enrollment and consent process.

Although head of the schools decide whether schools would take part in the trial, the final decision regarding participation is formed on an individual level for each student by their parents (Figure 2). To adhere to ethical and data protection regulations, both the adolescent and their parents (or legal custodians) must provide informed consent. Once the collaboration with the school is formally established, an information meeting for teachers and parents is scheduled. During this meeting, families can also ask questions in private and confidential settings. Subsequently, parents are asked to provide their initial consent for schools to share their personal contact information with the research team. Participant information and formal digital consent forms are sent to the parents via a secure national digital mailbox system (mit.dk and e‐Boks) in Denmark. Written consent is obtained from both parents/legal custody holders, as required under Danish law. If only one parent has legal custody, their signature alone is sufficient. Once secured, the research team administer study information and formal digital consent forms to the adolescent via mit.dk and e‐Boks. At this stage, class meetings of participating schools are scheduled. Adolescents aged 15 or older are invited to provide written consent after being offered the opportunity to ask questions regarding the project in private.

**FIGURE 3:**
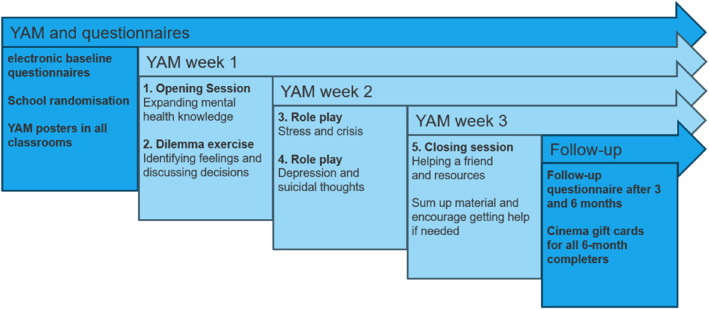
YAM research process.

Once the consent procedure is successfully completed, baseline data is collected electronically or on paper forms if the digital mailbox system is not installed for the adolescent (Figure 3). Data handling and storage complies with the European Union's General Data Protection Regulation (GDPR) (European Union 2016).

### 3.4 YAM Versus School Curriculum as Usual

The YAM program is a manualised, school‐based intervention designed to promote mental health awareness, increase help‐seeking behavior, and reduce risks of DSH and suicide among adolescents. The intervention consists of five sessions, which are delivered during school hours over 3‐to‐5 weeks (Carli et al. 2013). In the first session, students are introduced to core concepts in mental health literacy, including how to seek help for themselves or support a friend in need. Sessions two to four consist of interactive workshops that utilize dilemma cards and role‐playing exercises to explore emotional distress, social conflict, and coping mechanisms, all tailored to the student's preferences and everyday life experiences. In the final session, students are encouraged to reflect and reminded about mental health resources. Adolescents receive a 32‐page booklet, which covers topics such as stress, depression, self‐help, and supporting peers, and lists local community and national resources. Six educational posters are displayed in classrooms throughout the intervention to sustain engagement and promote awareness. Each session is delivered by two trained YAM instructors who are recruited based on previous work experiences with adolescents, preferably in teaching or healthcare. YAM instructors are certified through a 5‐day training seminar provided by the developers, Mental Health in Mind (MHiM). Instructors receive ongoing supervision from a certified child and adolescent psychiatrist and document delivery fidelity through structured logbooks submitted after each session (Carli et al. 2013).

Adolescents in the control group continue with their standard 8‐9th‐grade school curriculum. Additionally, the same six educational posters used in the intervention group are displayed in control classrooms for 3‐to‐5 weeks to ensure ethical equity.

### 3.5 Randomization and Blinding

Randomization is performed at the school level using a 1:1 allocation ratio. We opted for cluster randomization to prevent contamination between the intervention and control groups, as students often interact across classes within the same school. Given the nature of the intervention, it is not possible to blind either the students or the instructors. Data collectors and database managers are also aware of group assignments; however, the statistical analysis will be performed blinded.

### 3.6 Outcome Measures

#### 3.6.1 Feasibility Outcomes

Feasibility is assessed by three primary indicators (Table 1):

1. Participation rate: the percentage of invited adolescents and parents who provide informed consent.
2. Program adherence: the percentage of adolescents who attend at least four of the five YAM sessions.
3. Questionnaire completion: the percentage of adolescents who complete assessments at 3 and 6 months.

**TABLE 1:** Outcomes and data collection in the youth aware of mental health (YAM) feasibility trial.

Outcomes	Operational measure/scale	Baseline	3 months		6 months
Feasibility					
Program participation	Percentage of parents and adolescents consenting to participate		X		
Course completion	Percentage of adolescents attending the YAM classes (i.e., four or more of the total sessions)		X		
Response rates	Response rates on the questionnaire of participating adolescents		X		
Acceptability					
Adolescent endorsement	YAM questions regarding adolescents' endorsement		X		
Fidelity					
Yam intervention	A checklist of milestones on the delivery plan, which covers all steps from engaging with schools to the end of follow‐up		X		
Primary^a^					
Level of suicidal behavior	Paykel's suicidal scale (PSS)	X	X		X
Secondary^a^					
Well‐being index	WHO well‐being scale (WHO‐5)	X	X		X
Depression, anxiety, and stress score	Depression, anxiety, and stress scale (DASS21)	X	X		X
Help‐seeking intentions	General help‐seeking questionnaire (GHSQ)	X	X		X
Psychological distress	Kessler's psychological distress scale (K10)	X	X		X
Mental health knowledge and literacy	YAM‐specific scale	X	X		X
Stigma toward suicide	Stigma of suicide scale (SOSS)	X	X		X

^a^ Examined as explorative outcomes.

In addition to these overall indicators, school‐level variations in participation, adherence, and completion are descriptively assessed to identify potential barriers to engagement and inform future implementation planning.

#### 3.6.2 Acceptability and Fidelity Outcomes

Acceptability is assessed through self‐reports at the 3‐month follow‐up, where students enrolled in the YAM intervention indicate which sessions they have attended, that is the opening session, the dilemma card workshop, the role‐play sessions, and the final session. Fidelity is evaluated using a structured checklist assessing whether each planned session, activity, and instructional element is delivered as intended and any deviations from the manual are logged (Bailey et al. 2022). This checklist is completed by instructors and monitored by the research team.

#### 3.6.3 Exploratory Outcomes

The primary exploratory outcome is suicidal behavior, which captures both suicidal ideation and self‐harm acts and is measured using the Paykel Suicidal Scale (PSS) (Paykel et al. 1974). Secondary exploratory outcomes include:

- Mental well‐being, measured by the 5‐item WHO‐5 Well‐Being Index (WHO‐5) (Blom et al. 2012)
- Depression, anxiety, and stress, measured by the 21‐item short version Depression, Anxiety Stress Scales (DASS‐21) (Lovibond and Lovibond 1995)
- Psychological distress, measured by the 10‐item Kessler Psychological Distress Scale (K10) (Kessler et al. 2002)
- Help‐seeking intentions, measured by the 9‐item General Help‐Seeking Questionnaire (GHSQ) (Wilson et al. 2005)
- Mental health knowledge and literacy, measured by a 26‐item scale, previously developed and applied in YAM evaluations (D. Wasserman et al. 2015; Lindow et al. 2020b; McGillivray et al. 2021)
- Stigma related to suicide, measured by the 16‐item Stigma of Suicide Scale (SOSS) (Batterham et al. 2013)

These questionnaires are all validated and previously used in adolescent mental health research. All are administered electronically. Where Danish versions were not already available, questionnaires were translated using a standardized forward‐backwards translation procedure. Translated versions were reviewed linguistically by the research team and pilot‐tested for cultural relevance and comprehensibility among a small group of Danish adolescents before data collection.

### 3.7 Sample Size and Power Considerations

This feasibility pilot trial aims to inform the planning of a future full‐scale trial and is not powered to demonstrate efficacy of the YAM intervention. The trial's primary outcomes are feasibility indicators. Based on previous YAM investigations, we aim at a participation rate of around 28% among invited students, program adherence of around 85%, and response rates of around 67% (Carli et al. 2013; Lindow et al. 2020a). Based on the estimated number of participating schools and classes, we anticipate that 519 students will participate in the trial, with 345 completing the questionnaires.

### 3.8 Data Collection and Management

Data are collected at baseline and at 3‐ and 6‐month of follow‐up. Sociodemographic information (i.e., gender, age, country of birth, nationality, and parental relationship status) is collected at baseline. Adolescents complete the electronic questionnaires during school hours, with members of the research team present and available to answer questions and provide support as needed. Absent students are encouraged to complete the questionnaires via SMS reminders, with an offer of phone consultations. All data are recorded in REDCap, a secure electronic platform approved for research purposes in Denmark (REDCap, 2016), in accordance with GDPR compliance and Danish data protection law, and anonymized before analysis.

### 3.9 Statistical Analysis

Feasibility and acceptability outcomes will be summarized in numbers and percentages, both for the individual schools and the intervention groups in total. The exploratory outcomes will be analyzed using a multi‐level model that includes fixed effects of follow‐up time and the constrained time intervention group and random effects of school, class, and individual participant. Estimated treatment differences with 95% confidence intervals, variance components, and intra‐class correlations will be reported. All enrolled participants will be included in the analysis, regardless of whether they discontinued the study or adhered to the intervention. Numbers and reasons for missing data will be compared between the intervention groups using descriptive tables. Baseline characteristics of adolescents in each intervention group will be compared in descriptive tables. If a chance imbalance occurs between potential predictors of the outcomes, further post hoc exploratory analyses will be conducted while adjusting for their effects.

### 3.10 Safety Protocol

A standardized safety protocol is applied to all participating adolescents regardless of allocation (Table 2). Questionnaire responses are monitored through REDCap, where flags mark answers of concern (REDCap, 2016). All flagged responses are reviewed by the project's principal investigator, who has expertise in suicide risk assessment and evaluates the risk of the adolescents on a 1‐to‐4 scale (high to low priority). Adolescents with flagged responses are contacted by telephone by the research team contacts. Parents are contacted as a second step to ensure transparency and support. The research team includes a child and adolescent psychiatrist who is available for subacute and general supervision when needed. During the initial call, the research team conducts a risk assessment and determines whether the adolescent is already receiving care. When deemed necessary, adolescents are referred to school support staff or external professionals in accordance with existing legislation (§154 of the Danish Social Services Act). If a family member is at imminent risk, they are instructed to accompany the adolescent to the emergency department for clinical assessment and treatment. School nurses, educational psychologists, or other student support staff are available at all participating schools if needed, and their contact details are included in the YAM booklet.

**TABLE 2:** Safety procedures based on responses to suicidality and distress measures.

Paykel's suicidal scale	Actions initiated after the following responses:	Priority 1–4	Safety procedure
5‐Item questionnaire, response options: Yes/no			
Have you thought about taking your own life even if you weren't really going to? (item 3)	Yes	Priority 2	YAM team member takes contact with adolescent to follow‐up on response and conduct risk assessment and screening
Have you reached the point where you considered actually taking your own life or you made plans about how you would do it? (item 4)	Yes	Priority 1	(a) Call the adolescent (b) Call the parents (c) Conduct a risk assessment and screening (d) Initiate safety measures (e) Notify relevant parts
Have you tried to take your own life? (item 5)	Yes	Priority 1	(a) Call the adolescent (b) Call the parents (c) Conduct a risk assessment and screening (d) Initiate safety measures (e) Notify relevant parts

Kessler's psychological distress scale	Actions initiated after the following responses:	Priority 1–4	Safety procedure
10‐item likert scale, five response options: (1) Not at any point in time (2) A bit of the time (3) Some of the time (4) Most of the time (5) All of the time			
During the past 30 days, how often did you feel depressed? (item 7)	(4) Most of the time (5) All the time	Priority 3–4	YAM team member takes contact with adolescent to follow‐up on response and conduct risk assessment and screening
During the past 30 days, how often did you feel that everything was an effort? (item 8)	(4) Most of the time (5) All the time	Priority 3–4	YAM team member takes contact with adolescent to follow‐up on response and conduct risk assessment and screening
During the past 30 days, how often did you feel so sad that nothing could cheer you up? (item 9)	(4) Most of the time (5) All the time	Priority 3–4	YAM team member takes contact with adolescent to follow‐up on response and conduct risk assessment and screening
During the past 30 days, how often did you feel worthless? (item 10)	(4) Most of the time (5) All the time	Priority 3–4	YAM team member takes contact with adolescent to follow‐up on response and conduct risk assessment and screening

During the YAM sessions, any adolescent showing signs of distress is attended to by one of the instructors. Instructors are trained to respond to these situations and, when needed, alert the YAM research team, who coordinate with the student support staff. The YAM material lists referral options adapted to each participating school, including local psychologists, social services, and emergency contacts. Referral options are also included on the posters displayed in classrooms and included in the student booklet. The child and adolescent psychiatrist can supervise instructors as needed. Families are briefed on all safety procedures during the parental information meeting, as well as in the participant information material, where it is also stated that they should contact the nearest psychiatric emergency department in case of an acute crisis outside of school hours. Contact information is included in all YAM materials distributed to adolescents and their families.

## 4 Results

This study protocol for a feasibility trial is still ongoing. Recruitment began in August 2024. The intervention was given between November and December 2024, and the 6‐month follow‐up assessment is scheduled to end in July 2025. The study's results are published at a later stage.

## 5 Discussion

### 5.1 Study Design and Rationale

This two‐armed, cluster‐randomized feasibility pilot trial evaluates the delivery of the YAM program to 8‐9th‐grade students in Danish public schools, focusing on feasibility, fidelity, acceptability, and participation. The approach aligns with recommendations that a complex intervention targeting vulnerable populations should first be assessed for feasibility to inform whether and how it can be implemented effectively in a real‐world setting (Craig et al. 2008). By collecting data on deliverables, consent procedures, adherence, and adolescent self‐reported outcomes, this trial facilitates the identification of necessary adaptations and supports future planning for implementation.

The cluster randomization minimizes contamination between intervention and control conditions. This is important, especially in a school‐based trial where adolescents often interact across classes (Carli et al. 2013; Bailey et al. 2022). Furthermore, this allocation format is suitable because the YAM program is delivered to groups with peer interaction as a central component (D. Wasserman et al. 2015). Although blinding of adolescents and facilitators is not feasible, a researcher remains blinded during data analysis. This approach aligns with CONSORT recommendations and helps reduce the risk of confirmation bias in interpreting self‐reported outcomes (Eldridge et al. 2016).

This trial differs from previous feasibility evaluations of YAM conducted in the U.S. and Australia, which primarily focused on acceptability, mental health literacy, and stigma (Lindow et al. 2020b; McGillivray et al. 2021). In contrast, the current trial incorporates a structured safety protocol, systematic risk monitoring, and a detailed assessment of feasibility indicators, including participation rates, adherence, and fidelity. While YAM materials are typically adapted for local use, this study also incorporates context‐specific elements tailored to the Danish school system. Additionally, the use of a cluster‐randomized design at the school level ensures alignment with the program's universal delivery model.

### 5.2 YAM in Denmark

The Danish YAM intervention adheres to the key principles of structured, school‐based psychoeducational programming. The YAM instructors have relevant qualifications in education or healthcare and complete an intensive 5‐day training seminar provided by MHiM. This seminar ensures that instructors can deliver the program with fidelity and effectively respond to sensitive and emotional issues. Instructors work in pairs, maintain logbook documentation, and receive ongoing supervision, an approach shown to support program fidelity and responsiveness to adolescent needs (Bailey et al. 2022; Lindow et al. 2020a). Additionally, the flexible delivery window of 3‐to‐5 weeks allows schools to tailor schedules to their local logistics, increasing the feasibility of integrating YAM into the standard curriculum. Program adaptability has been identified as a key factor in the successful delivery of complex school environments, provided that core components remain intact (Domitrovich et al. 2010).

By embedding the program within public schools, the trial tests a low‐threshold intervention strategy, which can be scaled up cost‐effectively if proven feasible. Results from previous trials conducted in other cultural settings were mixed and underscored the need for feasibility evaluation prior to national implementation (D. Wasserman et al. 2015; Lindow et al. 2020b; McGillivray et al. 2021). Further, a systematic review and meta‐analysis on youth suicide prevention emphasized that universal school‐based programs should undergo context‐sensitive assessment of feasibility, fidelity, and population fit to ensure reliable evaluation of their effectiveness (Robinson et al. 2018).

### 5.3 Outcome and Safety Measures

In line with best practices for feasibility research, this trial prioritizes exploratory outcomes that capture key dimensions of adolescent mental health. Suicidal behavior, particularly suicidal ideation, was chosen as a key outcome due to its well‐documented association with later suicide attempts (O’Connor and Nock 2014; Reeves et al. 2022). Its often fluctuating nature, in severity, duration, and recurrence, underscores the importance of repeated measurement, which this trial addresses by including follow‐up assessments at 3 and 6 months. This approach enables a more nuanced understanding of short‐term changes and facilitates the identification of early intervention needs. The selection of the studied outcomes was guided by relevance to adolescent mental health and prior use in similar trials, ensuring transparency and consistency with established measures (Robinson et al. 2018). By incorporating these principles, the current trial aims to establish a methodologically sound foundation for informing outcome prioritization and power calculations in the event of a future full‐scale effectiveness trial.

This trial integrates a structured safety protocol to ensure ethical responsibility. Risk screening is conducted by the project's principal investigator and trained research assistants, who have expertise in suicide prevention. Adolescents identified as being at risk are contacted directly by the research team, with additional support provided in collaboration with a senior consultant from the Danish helpline for suicide prevention. Materials are adapted to include local crisis contacts and encourage help‐seeking through posters and student booklets. Together, these measures demonstrate that universal school‐based programs can be delivered with ethically grounded, proactive safeguards that promote adolescent psychological safety and meet established standards for suicide prevention research (Madge et al. 2008; Deisenhammer et al. 2009; Robinson et al. 2018).

### 5.4 Strengths and Limitations

This trial has strengths. The school‐level cluster‐randomized design mirrors the way universal interventions like YAM are typically implemented in educational settings, making it an appropriate and pragmatic choice (D. Wasserman et al. 2015). The trial draws on local infrastructure and aligns with existing educational and health systems by involving school nurses, educational psychologists, or other student support staff in logistical coordination, adaptation, and safety monitoring (Damschroder et al. 2022). Notably, the structured fidelity monitoring process and use of safety protocols for risk screening, referral, and follow‐up ensures responsible delivery and safeguarding of participating adolescents (Robinson et al. 2018).

Limitations should be acknowledged. The trial is not powered to detect clinically relevant differences in efficacy outcome However, anticipated participation rates suggest the study may provide tendencies or directions regarding a potential effect. Additionally, although the trial includes diverse public schools across several Danish regions, excluding private schools may limit the generalizability of the findings. Furthermore, the relatively small number of participating schools limits the generalizability of school‐level feasibility findings. This is due to practical constraints, including limited instructor capacity, as no previous Danish instructors have been certified, budget constraints, and the study's fixed timeframe. Self‐selection bias is another potential limitation, as adolescents or families with a particular interest in mental health may be more likely to participate (Madge et al. 2008). The lack of blinding of participants, instructors, and data collectors may introduce reporting biases albeit a blinded researcher conducts the statistical analyses. Finally, the self‐report outcome measures may be susceptible to underreporting or social desirability bias, especially concerning sensitive issues like suicide or self‐harm (Gillies et al. 2018).

## 6 Conclusion and Future Directions

This feasibility pilot trial provides information regarding the delivery, safety, and acceptability of the YAM program for 8‐9th‐grade students in Danish public schools. By assessing feasibility indicators, such as participation, fidelity, and completion, and also exploring changes in key self‐reported mental health outcomes, the study will inform recruitment strategies, refine procedures for fidelity monitoring, and support the selection and timing of outcome assessments in future trials. Additionally, by examining delivery processes and contextual factors in real‐world school settings, we will collect insights to inform the design and implementation of a full‐scale effectiveness trial. Findings from this first Nordic feasibility evaluation of YAM may help identify necessary adaptations and guide next steps toward evaluating the program's potential within broader school‐based mental health strategies.

## Author Contributions

**Lasse K. Harris:** writing – original draft, visualization, project administration. **Michella Heinrichsen:** writing – review and editing, project administration, investigation. **Marie Petri:** writing – review and editing, project administration, investigation, visualization, resources. **Hanne Elberling:** writing – review and editing, supervision. **Julie L. Forman:** writing – review and editing, formal analysis. **Merete Nordentoft:** conceptualization, methodology, funding acquisition, writing – review and editing, supervision. **Annette Erlangsen:** conceptualization, methodology, supervision, writing – review and editing, funding acquisition. **Britt R. Morthorst:** conceptualization, methodology, supervision, writing – review and editing, funding acquisition, investigation, visualization, project administration.

## Funding

The Novo Nordisk Foundation (NNF23SA0083854) funds the YAM feasibility trial in Denmark.

## Ethics Statement

This trial adheres to the principles outlined in the Declaration of Helsinki. The data collection was approved by the Regional Danish Data Protection Agency (P‐2023–14866). The study protocol was approved by the Regional Committee on Health Research Ethics (H‐24000117, amendment 115729). According to the National Committee on Health Research Ethics guidance, written informed consent is collected from legal guardians and participating adolescents.

## Conflicts of Interest

The authors declare no conflicts of interest.

## Data Availability

Data sharing not applicable to this article as no datasets were generated or analyzed during the current study.
